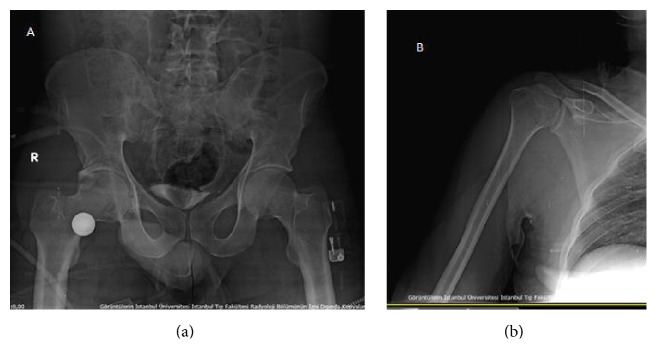# An Unusual Traumatic Presentation: Luxatio Erecta Humeri and Concomitant Hip Dislocation

**DOI:** 10.1155/2016/6910945

**Published:** 2016-12-19

**Authors:** Mehmet Demirel, Berkan Anarat, Mehmet Ersin, Ali Erşen, Cengiz Şen

**Affiliations:** Istanbul Faculty of Medicine, Department of Orthopaedics and Traumatology, Istanbul University, Istanbul, Turkey

## Abstract

*Introduction.* Inferior dislocation of the glenohumeral joint, known as luxatio erecta humeri, and posterior hip dislocation are both rare presentations in the emergency department. The most common aetiology is falling for luxatio erecta humeri. The aim of this manuscript was to present a unique case in terms of luxatio erecta humeri, which has a different aetiology, treatment method, and concomitant injury.* Presentation of Case.* We report a construction worker who was rescued from a collapsed building who presented with both luxatio erecta humeri and complex posterior hip dislocation. An orthopaedic surgeon reducted luxatio erecta humeri with a one-step reduction technique under procedural anaesthesia as soon as the patient's vital signs were stable.* Discussion.* Different concomitant injuries and various injury mechanisms have been described in regard to inferior shoulder dislocation in the literature. However, posterior dislocation of the hip as a concomitant distant region injury and trapping as an injury mechanism for luxatio erecta humeri are being described for the first time in this case report. Two reduction manoeuvers, one-step and two-step, have been used for this dislocation. Some authors suggested that a two-step manoeuver can be more easy to apply. In our specific case, luxatio erecta was easily reducted by a single operator in a single attempt.* Conclusion.* Luxatio erecta humeri may occur from trapping and complex injuries can accompany luxatio erecta humeri in patients with multiple trauma. A one-step closed reduction can be easily applied by a single operator under procedural anaesthesia.

## 1. Introduction

Inferior shoulder dislocation is an extremely rare disorder in the emergency department, accounting for 0.5% of all glenohumeral dislocations [1]. This rare dislocation may occur from a direct or indirect mechanism, the majority of which result from falling accidents [2, 3]. The first and most common mechanism is a direct loading force on a fully abducted arm, as seen on our case. The second mechanism is an indirect sudden forceful hyperabduction of an abducted upper extremity [2, 3].

Patient had also a concomitant hip dislocation. The aim of this article was to give an opinion regarding the conservative treatment of luxatio erecta humeri. In addition, we would like to share some experience about luxatio erecta humeri with an unusual mechanism and treatment of the injury.

## 2. Case

**Figure 1:**
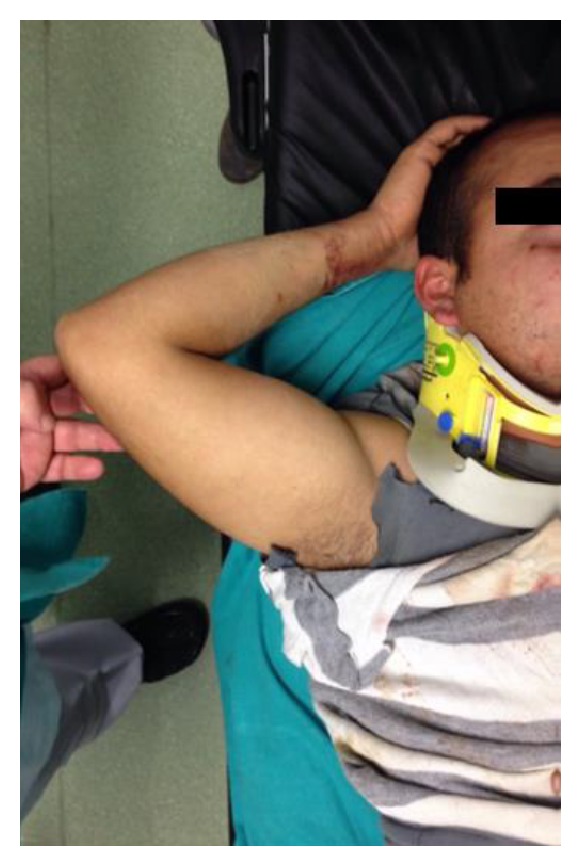


**Figure 2:**
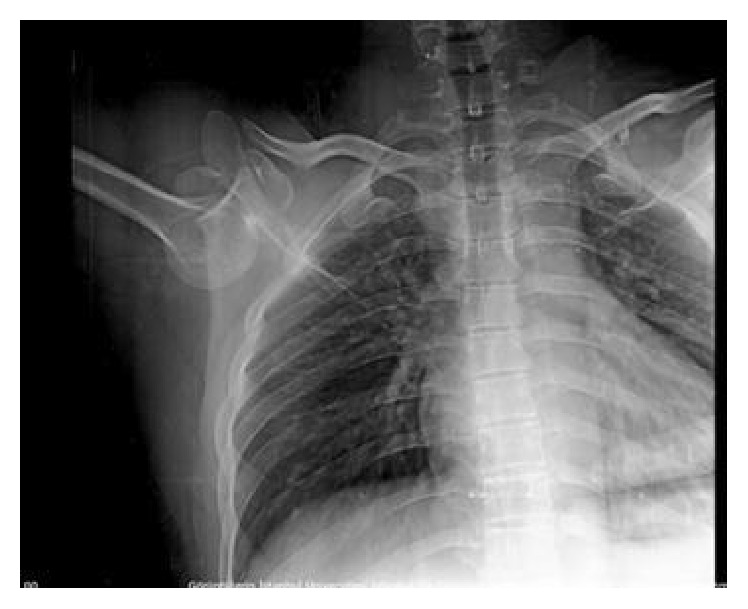


**Figure 3:**
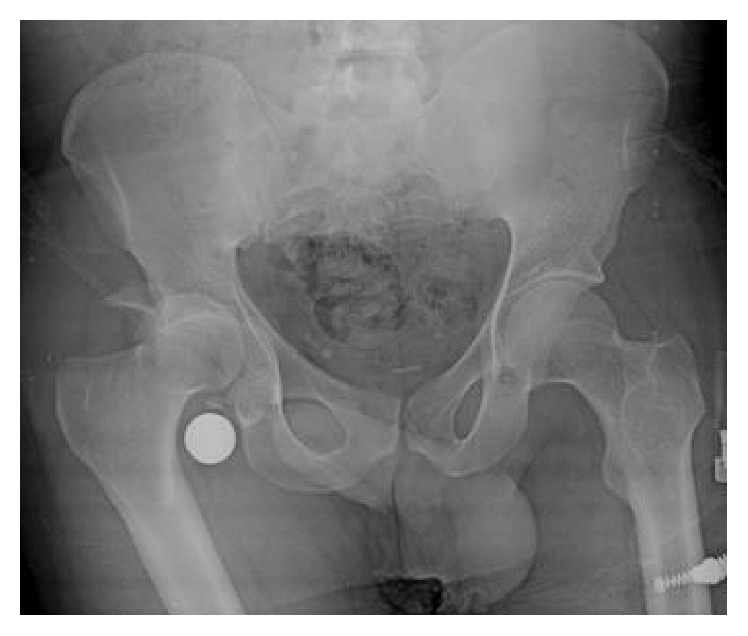


A man aged 28 years presented with his right upper extremity abducted at the shoulder, flexed at the elbow, pronated at the forearm, and with his hand behind his head (Figure 1). He was in a building collapse and was rescued 1 hour afterwards. Close questioning revealed the mechanism of his injury as direct axial loading force on a fully abducted upper extremity while he was protecting his body from the wreckage. His medical history was otherwise unremarkable and he was not using any medication. After his X-ray and computed tomography imaging, the diagnosis of luxatio erecta humeri (Figure 2) and complex posterior hip dislocation (Figure 3) was established. There was no fracture around the shoulder. However, a posterior wall fracture accompanied the posterior hip dislocation. As soon as the patient's vital signs were stable, an orthopaedic surgeon reducted the luxatio erecta humeri with a one-step reduction technique only under procedural anaesthesia in the emergency department (Figure 4). After closed reduction, the right shoulder was immobilized using a sling and skeletal traction was used to prevent recurrent dislocation of the hip. The patient was referred to our orthopaedics service after the emergency department. Treatment followed with internal fixation of the complex hip fracture dislocation on the 2nd day of his admission to hospital. In addition, the right arm was immobilized with a sling and the patient was reevaluated in 3rd week and 3rd and 12th month in outpatient clinic visits. In the last examination, a slight restricted range of motion in the hip and full range of motion in the shoulder were noted.

## 3. Discussion

Inferior shoulder dislocation, known as luxatio erecta humeri, was first defined by Middeldorph and Scharm in 1859 [4].

Gökkuş et al. reviewed 57 articles associated with the injury mechanism of all cases of luxatio erecta humeri, based on PubMed database. The authors reported that the most frequent mechanism was falling [3]. However, our patient was a construction worker who was rescued from a collapsed building; no other cases have been associated with the same mechanism, in either the review of Gökkuş et al. or the other studies. Our case is unique in respect of the injury mechanism.

Many concomitant fractures or fracture dislocations have been associated with inferior shoulder dislocation around the ipsilateral shoulder joint in the literature, such as avulsion fracture of the greater tuberosity, acromion fracture, clavicular fracture, acromioclavicular dislocation, fracture of the body of the scapula, glenoid fracture, and both forearm bone fractures [4–11]. Nevertheless, we encountered no cases of luxatio erecta humeri and concurrent hip dislocation. To our knowledge, this is the first case report to describe injuries of a distinct region other than the shoulder related to inferior shoulder dislocation. Also bilateral luxatio humeri cases were reported by Camarda et al. [12] and concomitant bilateral knee dislocation was reported by Foad and LaPrade [9].

Early reduction of inferior shoulder dislocation and hip dislocation is recommended to prevent complications [13, 14]. Closed reduction of either disorder may commonly be applied under procedural anaesthesia in the emergency room. Adequate sedation and analgesia or general anaesthesia have been described in the literature as essential to achieve a successful closed reduction [1, 2, 5, 11, 14]. Two reduction methods have been noted, two-step reduction and one-step traction-countertraction. The two-step reduction method was defined by Nho et al. as the specific reduction technique of inferior shoulder dislocations, which includes transforming the inferior dislocation to an anterior dislocation in order to reduce the joint to a suitable anatomic position before the ultimate reduction. The authors stated that this two-step reduction technique was easier than the traditional one-step traction-countertraction technique in many cases [4–11, 13–15].

In our case, we applied closed reduction with a one-step manoeuver without countertraction. The manoeuver was performed successfully with a single operator, in single attempt, and with minimal straight traction and adduction of the shoulder under the procedural anaesthesia. Immobilization and range of motion exercises resulted in full shoulder function at 3rd week and 3-month follow-up examinations. Closed reduction of inferior dislocation of the shoulder can be performed easily and comfortably with a one-step manoeuver by a single operator only under procedural anaesthesia, and it was comfortable for the operator and the patient. Additionally, we consider that the one-step manoeuver may be applied as easily as the two-step manoeuver, even by a single operator.

## 4. Conclusion

Luxatio erecta humeri may occur from trapping, depending on the direct loading force on a fully abducted arm. Different region injuries can accompany luxatio erecta humeri in a high energy trauma, as seen on our case. One-step closed reduction can be easily applied by a single operator and a single attempt without any sedation or anaesthesia, only under morphine analgesia. Therefore, we think that one-step reduction can be applied as easily as the two-step reduction technique.

## Figures and Tables

**Figure 4:**